# Characterization of Gastric Tissue-Resident T Cells in Autoimmune and *Helicobacter pylori*-Associated Gastritis

**DOI:** 10.3390/cimb44060167

**Published:** 2022-05-25

**Authors:** Daisuke Kametaka, Masaya Iwamuro, Takahide Takahashi, Araki Hirabata, Kenta Hamada, Yoshiyasu Kono, Hiromitsu Kanzaki, Seiji Kawano, Takehiro Tanaka, Fumio Otsuka, Yoshiro Kawahara, Hiroyuki Okada

**Affiliations:** 1 Department of Gastroenterology and Hepatology, Okayama University Graduate School of Medicine, Dentistry and Pharmaceutical Sciences, Okayama 700-8558, Japan; johnrambo851@yahoo.co.jp (D.K.); pidw8xq3@okayama-u.ac.jp (K.H.); hxnwq178@yahoo.co.jp (Y.K.); kanzaki@qc4.so-net.ne.jp (H.K.); skawano@mpd.biglobe.ne.jp (S.K.); hiro@md.okayama-u.ac.jp (H.O.); 2 Department of Gastroenterology, Iwakuni Clinical Center, 1-1-1 Atago-cho, Iwakuni, Yamaguchi 740-8510, Japan; 3 Division of Medical Support, Okayama University Hospital, Okayama 700-8558, Japan; taktak0611@gmail.com (T.T.); prhy0fft@gmail.com (A.H.); 4 Department of Pathology, Okayama University Graduate School of Medicine, Dentistry and Pharmaceutical Sciences, Okayama 700-8558, Japan; takehiro@md.okayama-u.ac.jp; 5 Department of General Medicine, Okayama University Graduate School of Medicine, Dentistry and Pharmaceutical Sciences, Okayama 700-8558, Japan; fumiotsu@md.okayama-u.ac.jp; 6 Department of Practical Gastrointestinal Endoscopy, Okayama University Hospital, Okayama 700-8558, Japan; yoshirok@md.okayama-u.ac.jp

**Keywords:** flow cytometry, autoimmune gastritis, atrophic gastritis, *Helicobacter pylori*

## Abstract

Data regarding the in-depth surface marker profiles of gastric tissue-resident lymphocytes in autoimmune and *Helicobacter pylori*-associated gastritis are lacking. In this study, we investigated potential differences in lymphocyte composition between these profiles. We enrolled patients with autoimmune (*n* = 14), active (current infection of *H. pylori* in the stomach; *n* = 10), and inactive gastritis (post-eradication of *H. pylori*; *n* = 20). Lymphocytes were isolated from the greater curvature of the stomach and lesser curvature of the body and analyzed using flow cytometry. The CD8^+^/CD3^+^ and CD4^+^/CD3^+^ ratios differed between the samples. Body CD4^+^/antrum CD4^+^, which is calculated by dividing the CD4^+^/CD3^+^ ratio in the body by that in the antrum, was significantly higher in autoimmune gastritis (3.54 ± 3.13) than in active (1.47 ± 0.41) and inactive gastritis (1.42 ± 0.77). Antrum CD8^+^/CD4^+^ in autoimmune gastritis (7.86 ± 7.23) was also higher than that in active (1.49 ± 0.58) and inactive gastritis (2.84 ± 2.17). The area under the receiver operating characteristic curve of antrum CD8^+^/CD4^+^ was 0.842, and the corresponding optimal cutoff point was 4.0, with a sensitivity of 71.4% and a specificity of 93.3%. We propose that an antrum CD8^+^/CD4^+^ ratio > 4.0 is a potential diagnostic marker for autoimmune gastritis.

## 1. Introduction

Autoimmune gastritis, also known as type A gastritis, is characterized by the destruction of oxyntic glands in the stomach, followed by the emergence of atrophic and metaplastic mucosa [1,2,3,4,5,6,7]. A significant reduction in the gastric glands results in vitamin B12 and iron malabsorption, leading to pernicious anemia and iron deficiency anemia. Neoplasms, including neuroendocrine tumors and gastric cancers, can occur in the stomach because of hypergastrinemia, chronic, persistent inflammation, and damage to the mucosa [8,9]. It is also known that patients with autoimmune gastritis often have other autoimmune disorders, most commonly an autoimmune thyroid disease [10]. As specific treatments for autoimmune gastritis have not been established, we lack measures to prevent the development and progression of inflammation or restore the damaged mucosa in patients with this affliction. However, a timely and accurate diagnosis of this disease is important because it allows the supplementation of vitamin B12 and/or iron and the surveillance of gastric neoplasms and concomitant autoimmune diseases.

The diagnosis of autoimmune gastritis largely depends on endoscopic examination, except for patients in the advanced phase presenting with pernicious anemia. The representative endoscopic features of autoimmune gastritis are fornix- and corpus-predominant atrophy, whereas atrophic changes start from the antrum and gradually progress to the proximal side in *Helicobacter pylori*-associated gastritis. However, the prompt diagnosis of autoimmune gastritis during esophagogastroduodenoscopy is sometimes difficult, owing to the appropriate judgment depending on the subjective interpretation of endoscopy images and the expertise level of the endoscopists involved. In addition, the identification of fornix- and corpus-predominant atrophy may be difficult when *H. pylori*-associated gastritis and autoimmune gastritis coexist. Thus, since autoimmune gastritis is frequently misdiagnosed or overlooked in real-world clinical practice [11], the establishment of a fast and objective diagnostic tool is required.

Flow cytometry enables an objective estimation of cell numbers based on the presence of specific cell surface markers. In our earlier study, we established a one-step lymphocyte isolation procedure from endoscopically biopsied gastrointestinal mucosa [12,13,14]. We initially used this procedure to diagnose gastrointestinal lymphoma [15] and subsequently investigated lymphocytes in gastrointestinal diseases. For instance, flow cytometric analysis of peripheral blood mononuclear cells and colorectal mucosa lymphocytes in patients with ulcerative colitis and control individuals revealed that central memory T lymphocytes (CD45RA^−^CD62L^+^ T-cell subset) are increased, whereas natural killer T cells (CD56^+^/CD3^+^ subset) are decreased in the rectum of patients with ulcerative colitis [16]. These results indicate that flow cytometry can characterize disease-specific T lymphocytes and may serve as a diagnostic tool for disease discrimination. Based on the findings of our previous studies, we performed a comprehensive flow cytometric characterization of T lymphocytes in patients with autoimmune gastritis, current *H. pylori*-associated gastritis (active gastritis), and atrophic gastritis after *H. pylori* eradication (inactive gastritis). The primary purpose of this study was to reveal the distinctive features of lymphocytes that can be used as diagnostic markers for differentiating autoimmune gastritis from *H. pylori*-associated gastritis.

## 2. Results

### 2.1. Patient Characteristics

The clinical characteristics of the enrolled patients are summarized in Table 1. In the autoimmune gastritis group, there were five men and nine women. The average age was 63.2 years (range: 40–78 years). The details of each patient in the autoimmune gastritis group are shown in Table 2. Anti-parietal cell antibody was positive in 13 patients (92.9%), and the anti-intrinsic factor antibody was positive in four patients (28.6%). Serum gastrin levels were measured in 11 patients and found to be elevated in all patients (100%, reference range: 42–200 pg/mL). Serum vitamin B12 levels were lower in 7/14 patients (50.0%, reference range: 233–914 pg/mL), whereas folic acid levels were within the normal range in all patients in whom it was measured (12/12: 100%; reference range: 3.6–12.9 ng/mL). Serum iron levels were decreased in two patients (2/14: 14.3%; reference range: 54–200 μg/dL in men and 48–154 μg/dL in women). Serum pepsinogen I and II levels were evaluated in 12 patients. Pepsinogen I and I/II ratios were lower in all patients. With respect to *H. pylori* infection status, eight patients were uninfected, whereas the previous infection of *H. pylori* was confirmed in the remaining six patients. Five of the patients had gastric neoplasms, including adenoma (*n* = 2), neuroendocrine tumor G1 (carcinoid) (*n* = 2), and early gastric cancer confined to the mucosal layer (*n* = 2, well-differentiated adenocarcinoma in one patient and signet ring cell carcinoma in the other patient). In one patient, adenoma and well-differentiated adenocarcinoma developed asynchronously in the stomach.

The active gastritis group consisted of 10 patients with a current *H. pylori* infection, including five men and five women. Their average age was 64.1 years (range: 43–78 years). As described in Section 4, the datasets of 20 patients with atrophic gastritis after eradicating *H. pylori*, which were acquired in our earlier work [17], were used as the inactive gastritis group. This study included 10 men and 10 women. The average age was 68.5 years (range: 44–82 years). No statistical differences were observed in terms of age among the three groups. Furthermore, significant differences were not observed in terms of sex between the three groups, despite female predominance in the autoimmune gastritis group (9/14,: 64.3%).

### 2.2. Lymphocyte Composition in Patients with Autoimmune, Active, and Inactive Gastritis Patients

**Figure 1:**
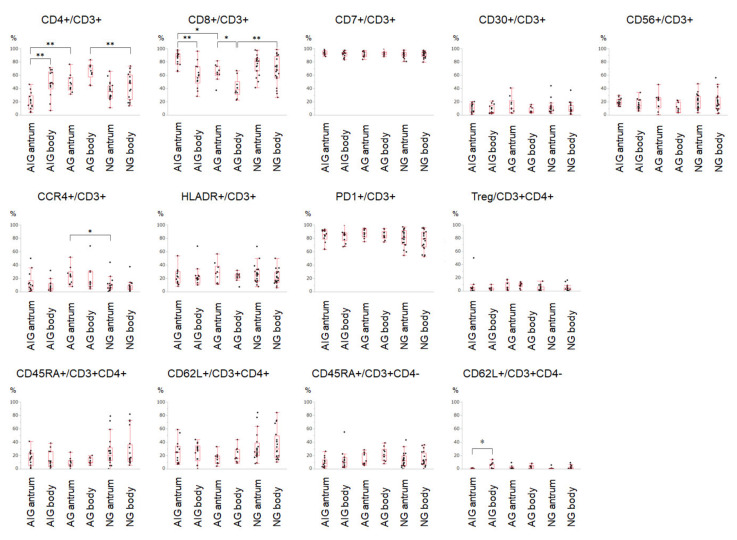
Box plots of flow cytometry results of each group. * *p* < 0.05; ** *p* < 0.01; AIG: autoimmune gastritis (*n* = 14); AG: active gastritis (*n* = 10); NG: inactive gastritis (*n* = 20); Tregs: regulatory T cells, defined as CD3^+^CD4^+^CD25^+^CD12^7low/−^ cells.

The flow cytometry results for all samples are shown in Figure 1. A comparison between the greater curvature of the antrum and lesser curvature of the body samples in the autoimmune gastritis group revealed that the CD8^+^/CD3^+^ ratio was elevated in the antrum compared to that in the body (85.0 ± 10.3% vs. 58.9 ± 18.3%). Conversely, the CD4^+^/CD3^+^ ratio was lower in the antrum than in the body (19.7 vs. 13.1% vs. 47.7 ± 19.3%). The CD62L^+^/CD3^+^CD4^−^ ratio was also lower in the antrum (0.34 ± 0.55% vs. 3.4 ± 4.5%). The expression of other surface markers investigated in this study did not differ between the stomach locations in patients with autoimmune gastritis. In active gastritis, the CD8^+^/CD3^+^ ratio was elevated in the antrum compared to that in the body (63.7 ± 12.3% vs. 40.8 ± 14.6%). In patients with inactive gastritis, no difference was observed in lymphocyte composition between the greater curvature of the antrum and lesser curvature of the body samples.

A comparison of the lymphocytes in the greater curvature of the antrum samples between the groups revealed that the CD4^+^/CD3^+^ ratio was lower in autoimmune gastritis group members than that in the active gastritis group (19.7 ± 13.1% vs. 47.5 ± 13.5%). In contrast, the CD8^+^/CD3^+^ ratio was elevated in the autoimmune gastritis group participants compared to the active gastritis group (85.0 ± 10.3% vs. 63.7 ± 12.3%). The CCR4^+^/CD3^+^ ratio in the antrum was higher in the active gastritis group than in the inactive gastritis group (23.4 ± 13.4% vs. 9.6 ± 9.8%).

Lymphocytes of the lesser curvature of the body showed that the CD4^+^/CD3^+^ ratio was higher in the active gastritis group than in the inactive gastritis group (66.1 ± 12.8% vs. 43.8 ± 19.4%). The CD8^+^/CD3^+^ ratio was lower in the active than in the inactive gastritis group (40.8 ± 14.6% vs. 67.1 ± 21.3%).

### 2.3. Potential Autoimmune Gastritis Diagnostic Marker

**Figure 2:**
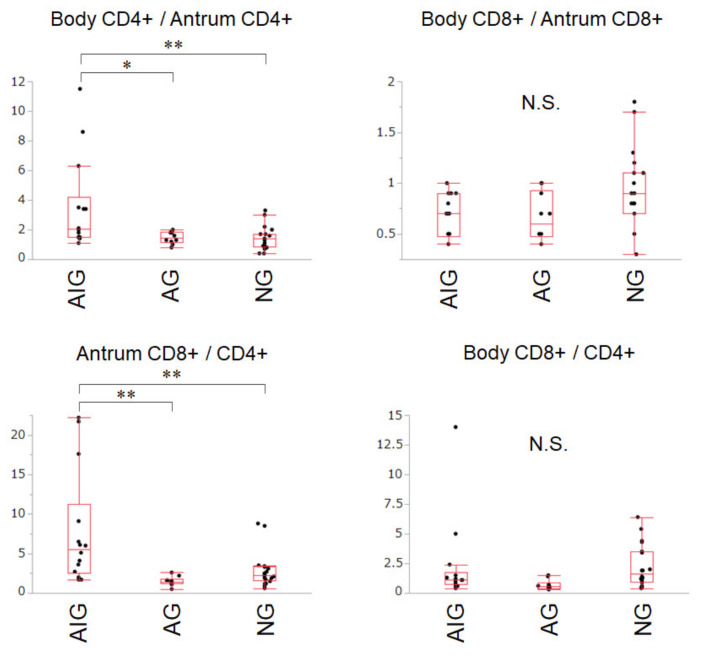
Box plots of the four potential markers combining CD4^+^/CD3^+^ and CD8^+^/CD3^+^ ratios in the greater curvature of the antrum and those of the lesser curvature of the body. * *p* < 0.05; ** *p* < 0.01; AIG: autoimmune gastritis (*n* = 14); AG: active gastritis (*n* = 10); NG: inactive gastritis (*n* = 20); N.S.: no significant differences.

Based on the differences in lymphocyte composition between the groups, we focused on CD4^+^ and CD8^+^ lymphocytes. We identified four potential diagnostic markers for autoimmune gastritis by combining CD4^+^/CD3^+^ and CD8^+^/CD3^+^ ratios in the greater curvature of the antrum and those of the lesser curvature of the body. As shown in Figure 2, body CD4^+^/antrum CD4^+^, which is calculated by dividing CD4^+^/CD3^+^ in the lesser curvature of the body by CD4^+^/CD3^+^ in the greater curvature of the antrum, was significantly higher in autoimmune gastritis (3.54 ± 3.13) than in active (1.47 ± 0.41) and inactive gastritis (1.42 ± 0.77). Antrum CD8^+^/CD4^+^ of autoimmune gastritis (7.86 ± 7.23), calculated by dividing CD8^+^/CD3^+^ by CD4^+^/CD3^+^ in the greater curvature of the antrum, was also higher than that of active (1.49 ± 0.58) and inactive gastritis (2.84 ± 2.17). Meanwhile, body CD8^+^/antrum CD8^+^ and body CD8^+^/CD4^+^ ratios did not differ between the groups.

**Figure 3:**
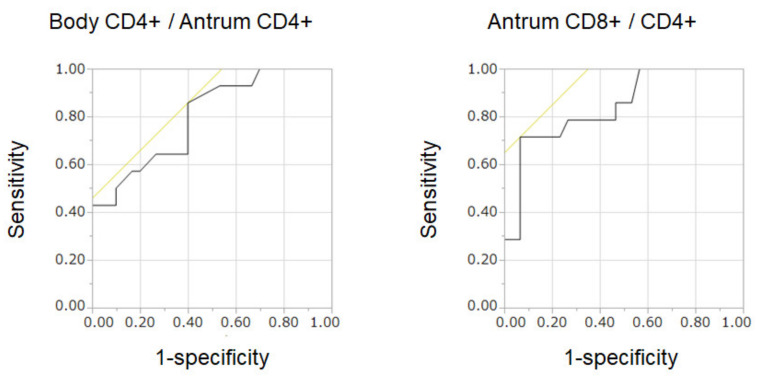
ROC curves: the area under the ROC curve of body CD4^+^/antrum CD4^+^ was 0.799, and that of antrum CD8^+^/CD4^+^ was 0.842.

The area under the receiver operating characteristic (ROC) curve of body CD4^+^/antrum CD4^+^ was 0.799, and that of antrum CD8^+^/CD4^+^ was 0.842 (Figure 3), indicating that the latter formula was superior to the former for the identification of autoimmune gastritis versus active or inactive gastritis. The corresponding optimal cutoff point of antrum CD8^+^/CD4^+^ was 4.0, with a sensitivity of 71.4% and a specificity of 93.3%.

## 3. Discussion

Comprehensive flow cytometry analysis of autoimmune, active, and inactive gastritis revealed that the lymphocyte composition differed in CD4^+^/CD3^+^ and CD8^+^/CD3^+^ ratios. We further proposed an antrum CD8^+^/CD4^+^ ratio > 4.0 as a potential diagnostic marker for autoimmune gastritis. To our knowledge, this report is the first to explore the detailed lymphocyte composition in autoimmune gastritis and *H. pylori*-associated gastritis and to investigate a flow cytometry-aided method of the objective distinction between them. In contrast to conventional subjective and operator-dependent diagnosis based on endoscopic and histopathological features of the stomach mucosa, we considered that diagnostic markers using flow cytometry would be a superior screening method to identify patients with autoimmune gastritis, as flow cytometry provides a rapid, objective, and quantitative recording of individual cells.

Despite the well-known features of corpus-predominant atrophy in autoimmune gastritis, the CD8^+^/CD4^+^ ratio in the gastric body (body CD8^+^/CD4^+^) did not differ between autoimmune, active, and inactive gastritis (Figure 3), whereas that of the gastric antrum (antrum CD8^+^/CD4^+^) represented a potential diagnostic marker. These results indicated that biopsy of the greater curvature of the antrum, rather than the lesser curvature of the body, may be valuable in identifying patients with autoimmune gastritis.

We speculate that the affected area of the stomach caused the difference in antrum CD8^+^/CD4^+^, rather than body CD8^+^/CD4^+^, between the gastritis types. Both the gastric body and antrum can be affected in *H. pylori*-associated gastritis, whereas the gastric antrum is not involved in autoimmune gastritis. CD4^+^ lymphocytes are reported to be mediators of inflammation in *H. pylori*-associated gastritis [18]. CD4^+^ lymphocytes also play a central role in the pathogenesis of autoimmune gastritis in patients and mouse models [19,20]. For instance, TxA23 mice, which express a transgenic T-cell receptor against the H+/K+ adenosine triphosphatase α chain, have been used as a mouse model of autoimmune gastritis [21,22]. Autoreactive inflammation against parietal cells is induced by CD4^+^ T cells in TxA23 mice, resulting in atrophic gastritis and metaplasia, ultimately leading to gastric cancer. Therefore, it is reasonable to believe that CD4^+^ lymphocytes are increased in both the body and antrum samples in *H. pylori*-associated gastritis, whereas they are increased in the body samples alone in autoimmune gastritis.

An analysis combining the lymphocyte composition of the antrum with that of the body revealed that the body CD8^+^/antrum CD8^+^ ratio was not different between the groups (Figure 3), whereas the body CD4^+^/antrum CD4^+^ ratio in autoimmune gastritis was higher than that in active and inactive gastritis. These results confirm that CD4^+^ lymphocytes mainly contribute to inflammation. It is also suggested that the number of CD4^+^ lymphocytes in the gastric body mucosa is larger than that in the antrum mucosa in autoimmune gastritis, whereas the gastric body and antrum are evenly affected by CD4^+^ lymphocyte infiltration in *H. pylori*-associated gastritis. Therefore, the difference in body CD4^+^/antrum CD4^+^ cells can be explained by the difference in the involved area in the stomach.

Although there were distinct differences in CD4^+^ and D8^+^ lymphocyte compositions, CD7^+^, CD30^+^, CD56^+^, CCR4^+^, HLADR^+^, PD1^+^, CR45RA^+^, and CD62L^+^ lymphocytes and Treg cells did not differ between autoimmune, active, and inactive gastritis. The present results indicate that inflammatory response processes might be largely shared among gastritis-causing mucosal atrophy despite different inflammatory stimuli, that is, autoantibodies against parietal cells and inflammatory responses against *H. pylori* infection. However, we cannot elucidate the roles of inflammatory cells in each disease based on the results of the present study, as this would be beyond the scope of our research. Thus, the differences in the inflammatory response between autoimmune, active, and inactive gastritis should be investigated.

Our study had certain limitations. First, all patients with autoimmune gastritis were diagnosed before enrollment in this study. Thus, the usefulness of antrum CD8^+^/CD4^+^ should be investigated in a population including patients with gastric atrophy of unknown etiology. A second limitation is the small number of participants. Due to the infrequency of the disease, we enrolled as many patients with autoimmune gastritis who were to undergo esophagogastroduodenoscopy at our institution as possible. For patients with *H. pylori*-associated gastritis, we excluded factors that might affect the lymphocyte composition of the gastric mucosa, such as inflammatory bowel disease, autoimmune gastritis, immunosuppressive or anticancer drugs, and previously existing gastric cancers. Thus, we believe that patient uniformity is warranted despite the small number of patients. Third, the autoimmune gastritis group included patients uninfected with *H. pylori* and those with previous *H. pylori* infection. However, we found several significant differences in lymphocyte composition despite the small sample size and diverse backgrounds of the enrolled patients.

In conclusion, our study showed that CD4^+^/CD3^+^ and CD8^+^/CD3^+^ ratios in the greater curvature of the gastric antrum and the lesser curvature of the body were different in autoimmune, active, and inactive gastritis. In addition, we proposed antrum CD8^+^/CD4^+^ > 4.0 as a potential diagnostic marker for autoimmune gastritis, with a sensitivity of 71.4% and a specificity of 93.3%. In our flow cytometry protocol, we used a single biopsy specimen from the gastrointestinal mucosa for lymphocyte isolation [12,13]. Thus, biopsy of a single specimen from the gastric antrum and flow cytometric analysis may represent a promising and objective diagnostic marker to identify autoimmune gastritis in patients with gastric mucosal atrophy. Such timely and accurate identification will enable earlier intervention in patients with autoimmune gastritis, including supplementation with vitamin B12 and/or iron as well as the surveillance of gastric neoplasms and concomitant autoimmune diseases.

## 4. Materials and Methods

### 4.1. Diagnosis of Autoimmune Gastritis, Active Gastritis, and Inactive Gastritis

All patients with autoimmune gastritis were diagnosed before enrollment in the study. Diagnosis of autoimmune gastritis was made based on (i) positive results for either anti-parietal cell or anti-intrinsic factor antibodies, (ii) pathologically confirmed corpus-predominant atrophy, and (iii) presence of endocrine cell hyperplasia upon microscopy.

Active gastritis was defined as a current infection of *H. pylori* in the stomach. *H. pylori* infection status was determined using urea breath tests, rapid urease tests, microscopic observations, culture tests of endoscopically biopsied specimens, serum antibody tests, or a combination of these methods.

Inactive gastritis was defined as atrophic gastritis after the eradication of *H. pylori*. All patients had a known history of *H. pylori* eradication, and eradication completion was previously confirmed using urea breath tests and microscopic observations of endoscopically biopsied specimens [17].

The inclusion criteria for active and inactive gastritis were as follows: (i) patients who had never undergone surgical resection of the stomach; (ii) patients who had never undergone gastric cancer treatment; (iii) patients who were not taking immunosuppressive or anticancer drugs; (iv) patients without known autoimmune gastritis or inflammatory bowel disease.

### 4.2. Biopsy Sample Acquisition and Flow Cytometry

**Figure 4:**
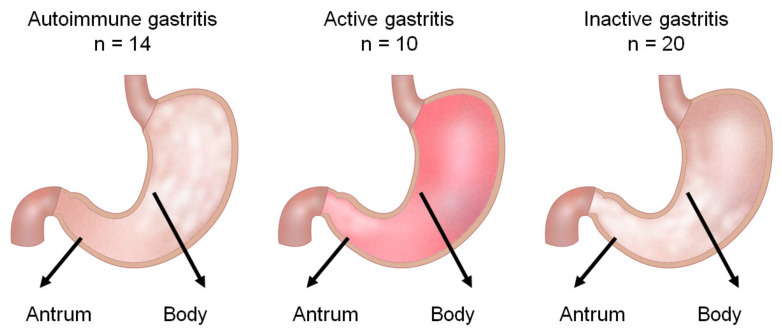
Schema of biopsy sample acquisition for flow cytometry. We endoscopically harvested one specimen from the greater curvature of the gastric antrum and the other specimen from the lesser curvature of the body.

To characterize gastric lymphocyte surface markers and reveal the distinctive features of lymphocytes in autoimmune, active, and inactive gastritis, we endoscopically harvested two specimens: one from the greater curvature of the gastric antrum and the other from the lesser curvature of the body (Figure 4). Lymphocytes were isolated from each biopsied specimen using a one-step lymphocyte isolation procedure, as reported in our earlier work [17].

Flow cytometry was prospectively performed at Okayama University Hospital (Okayama, Japan) between October 2020 and December 2021 on endoscopic biopsy specimens obtained from the gastric mucosa of 14 and 10 patients with autoimmune and active gastritis, respectively. In addition, we used flow cytometric data of 20 patients with inactive gastritis obtained in our earlier study [17], which were collected at the Okayama University Hospital between June 2020 and December 2020. Endoscopy and biopsy were performed as part of the standard care for neoplasia screening, mostly on a yearly basis, in all patients enrolled in the present study.

Lymphocytes isolated from the gastric mucosa were used for flow cytometry. To comprehensively analyze the profiles of T lymphocytes in patients with autoimmune gastritis, active gastritis, and inactive gastritis, monoclonal antibodies against CD45 (clone J33; Beckman Coulter, Pasadena, CA, USA), CD3 (UCHT1, Beckman Coulter), CD4 (13B8.2; Beckman Coulter), CD8 (B9.11; Beckman Coulter), CD7 (8H8.1; Beckman Coulter), CD25 (B1.49.9; Beckman Coulter), CD30 (HRS4; Beckman Coulter), CD45RA (2H4; Beckman Coulter), CD56 (N901; Beckman Coulter), CD62L (DREG56; Beckman Coulter), CD127 (R34.34; Beckman Coulter), CCR4 (i.e., CD194; L291H4; BioLegend, San Diego, CA, USA), HLA-DR (Immu-357; Beckman Coulter), and PD-1 (CD279; PD1.3; Beckman Coulter) were employed. Immunostained cells were analyzed using FACScan (Navios flow cytometer, Beckman Coulter) and Kaluza analysis software (v.1.3; Beckman Coulter). Lymphocytes were separated by flow cytometry based on high CD45 antigen expression and forward- and side-scatter properties. Subsequently, flow cytometric data were analyzed according to the percentage of cell populations detected in each quadrant on two-dimensional scatterplots. We calculated the percentages of CD4^+^, CD8^+^, CD56^+^, CD7^+^, PD1^+^, CCR4^+^, CD30^+^, and HLADR^+^ cells among the CD3^+^ cells. We also assessed the percentages of Treg, CD45RA^+^, and CD62L^+^ cells among CD3^+^CD4^+^ cells and the percentages of CD45RA^+^ and CD62L^+^ cells among CD3^+^CD4^−^ cells. In this study, we defined CD3^+^CD4^+^CD25^+^CD127^low/−^ cells as Tregs.

### 4.3. Analysis

First, to assess differences between lymphocyte populations in patients with autoimmune gastritis, active gastritis, and inactive gastritis, we compared flow cytometric results of stomach samples between the three groups. Second, we selected and combined several surface markers based on the results of the first analysis and developed discriminating methods to establish a potential diagnostic marker for autoimmune gastritis.

Statistical analyses were performed using JMP (v.14.0.0; SAS Institute Inc., Cary, NC, USA). The Student’s *t*-test or *F*-test was used to compare two population means. For multiple comparisons, statistical analysis was performed using a one-way analysis of variance followed by the Tukey–Kramer post hoc test. Statistical significance was set at *p* < 0.05. Numerical values are presented as means ± standard deviation.

### 4.4. Ethics Approval

Patients with autoimmune and active gastritis were prospectively registered and analyzed in this study. Written informed consent was obtained from all participants. Written informed consent was also obtained from patients with inactive gastritis to enable the reuse of flow cytometry datasets obtained in our earlier study [17]. This study adhered to the principles of the Declaration of Helsinki and was approved by the ethics committee of the Okayama University Hospital. The study protocol was registered with the UMIN Clinical Trials Registry (UMIN000041984).

## Figures and Tables

**Table 1:** Patient characteristics of autoimmune, active, and inactive gastritis.

	Autoimmune Gastritis	Active Gastritis	Inactive Gastritis
Age, mean ± SD, years (range)	63.2 ± 13.8 (40–78)	64.1 ± 13.1 (43–78)	68.5 ± 10.6 (44–82)
Sex			
Men	5	5	10
Women	9	5	10

**Table 2:** Details of the patients with autoimmune gastritis.

No.	Age	Sex	Anti-Parietal Cell Antibody	Anti-Intrinsic Factor Antibody	Gastrin (pg/mL)	Vitamin B12 (pg/mL)	Folic Acid (ng/mL)	Iron (μg/dL)	Pepsinogen I (ng/ml)	Pepsinogen I/II Ratio	*H. pylori* Infection
1	67	F	80	Negative	5964	120	17.35	76	5.6	0.6	Uninfected
2	64	F	>160	Positive	2300	419	9.8	85	21.6	1.4	Uninfected
3	43	M	40	Negative	2374	369	NA	63	7.4	1	Eradicated
4	73	M	20	Negative	NA	<100	16.9	94	6.3	0.6	Eradicated
5	73	M	10	±	3237	<100	NA	92	4	0.8	Uninfected
6	45	F	80	Negative	3787	1038	9.74	125	6.2	0.6	Uninfected
7	48	F	>160	Negative	NA	394	10.5	102	28.7	1.1	Eradicated
8	40	F	80	Negative	4714	123	8.26	29	NA	NA	Uninfected
9	53	F	>160	Negative	1167	238	14.2	64	3	0.3	Uninfected
10	73	F	>160	Negative	1448	1758	11.4	108	5.8	0.8	Eradicated
11	69	M	Negative	Positive	1484	49	8.8	52	NA	NA	Uninfected
12	78	F	40	Positive	NA	<100	13.9	224	3.7	0.3	Eradicated
13	73	M	10	Negative	9900	321	17.86	125	3.5	0.6	Uninfected
14	77	F	40	Positive	785	114	13.4	57	5.1	1	Eradicated

NA: not available.

## Data Availability

The data supporting the findings of this study are available from the corresponding author upon reasonable request. The data are not publicly available because they contain information that might compromise the privacy of the research participants.
